# Lumen charge governs gated ion transport in β-barrel nanopores

**DOI:** 10.1038/s41565-025-02052-6

**Published:** 2025-11-11

**Authors:** Simon Finn Mayer, Marianna Fanouria Mitsioni, Paul Robin, Lukas van den Heuvel, Nathan Ronceray, Maria Jose Marcaida, Luciano A. Abriata, Lucien F. Krapp, Jana S. Anton, Sarah Soussou, Justin Jeanneret-Grosjean, Alessandro Fulciniti, Alexia Möller, Sarah Vacle, Lely Feletti, Henry Brinkerhoff, Andrew H. Laszlo, Jens H. Gundlach, Theo Emmerich, Matteo Dal Peraro, Aleksandra Radenovic

**Affiliations:** 1 Institute of Bioengineering, School of Engineering, Swiss Federal Institute of Technology Lausanne, Lausanne, Switzerland; 2 Institute of Bioengineering, School of Life Sciences, Swiss Federal Institute of Technology Lausanne, Lausanne, Switzerland; 3 Institute of Science and Technology Austria, Klosterneuburg, Austria; 4 Department of Physics, University of Washington, Seattle, USA WA; 5 Laboratoire de Physique, UMR CNRS 5672, ENS de Lyon, Université de Lyon, Lyon, France

**Keywords:** Nanopores, Electrical and electronic engineering, Bionanoelectronics

## Abstract

β-Barrel nanopores are involved in crucial biological processes, from ATP export in mitochondria to bacterial resistance, and represent a promising platform for emerging sequencing technologies. However, in contrast to ion channels, the understanding of the fundamental principles governing ion transport through these nanopores remains largely unexplored. Here we integrate experimental, numerical and theoretical approaches to elucidate ion transport mechanisms in β-barrel nanopores. We identify and characterize two distinct nonlinear phenomena: open-pore rectification and gating. Through extensive mutation analysis of aerolysin nanopores, we demonstrate that open-pore rectification is caused by ionic accumulation driven by the distribution of lumen charges. In addition, we provide converging evidence suggesting that gating is controlled by electric fields dissociating counterions from lumen charges, promoting local structural deformations. Our findings establish a rigorous framework for characterizing and understanding ion transport processes in protein-based nanopores, enabling the design of adaptable nanofluidic biotechnologies. We illustrate this by optimizing an aerolysin mutant for computing applications.

## Abstract (web summary)

By unifying data from engineered β-barrel nanopores and supported by modelling, it is demonstrated that the lumen charge in a β-barrel nanopore governs rectification and voltage-driven gating, with applications in computing using nanofluidic synapses.

## Main

Pore-forming proteins are biological nanofluidic channels enabling the regulated or free passage of molecules and ions across membranes. They are essential for information transmission, import and export, as well as attack and defence of cells and organisms^1^. Out of their biological context, some pore-forming proteins have been repurposed as sensors for probing a diverse range of molecules^2–9^. These sensors are known as biological nanopores and have been commercialized for low-cost, long-read DNA sequencing^8^, a first demonstration of their potential for societal impact. Despite evident technological interest, ionic motion through biological nanopores still awaits proper rationalization. It is well known that ion transport through such biological channels is highly nonlinear^10,11^. In particular, the ionic current through such pores can be polarity dependent, an effect known as rectification^12–14^, and it can abruptly decrease, a phenomenon referred to as gating^11,15–23^. The mechanisms underlying such nonlinearities, particularly the role of lumen charge, remain poorly understood, hindering further progress in biological nanopore sensing technologies. This contrasts with solid-state nanofluidics, where advances in nanofabrication and theoretical modelling have enabled in-depth rationalization of ion transport in confinement^24^ and with ion channels, where decades of advances in molecular biology have shed light on a rich mechanistic phenomenology^10^. In biological nanopores, such a characterization is challenging owing to their heterogeneous surface charge, and their intermediate size around 1 nm, which precludes a strictly molecular or continuous understanding. Rationalizing ion transport in these pores is nonetheless of paramount importance owing to their pivotal role in living organisms as well as their potential for disruptive technology.

In this work, we aim to rationalize ion transport in biological pores, taking into account the complexity of their lumen charge as well as their mechanical properties. We focus on multimeric β-barrel-forming nanopores widely used in the nanopore field (Supplementary Fig. 2), such as the toxins aerolysin and α-haemolysin (α-HL), as well as *Mycobacterium smegmatis* porin A (MspA). They are to be distinguished from smaller highly specific ion channels that often undergo conformational changes to enable or disable transport of a specific species of ion^10^. To capture the complexity of the lumen charge, we study the dependence of gating on 26, 6 and 5 different mutants of aerolysin, α-HL and MspA, respectively. Such pores have constriction diameters between around 1 nm and 1.4 nm and a length of roughly 10 nm (refs. ^9,25^). Our study combines single-pore and ensemble transmembrane ion transport measurements, biophysical modelling, atomistic molecular dynamics (MD) simulations and cryo-electron microscopy (cryo-EM) characterization. We show that charge distribution in the pore lumen governs rectification and voltage-driven mechanical gating, we develop analytical models of these behaviours, and we harness this knowledge to design an aerolysin mutant with enhanced synaptic plasticity.

## Nonlinear responses of biological nanopores (rectification and gating)

**Fig. 1:**
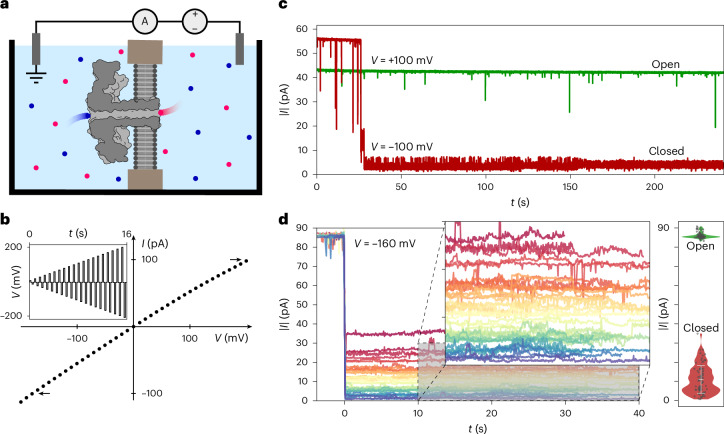
Complexity and diversity of ion transport in single aerolysin pores. **a**, Not-to-scale schematic of the experimental set-up. A lipidic membrane separating two reservoirs filled with electrolyte (cations are shown in blue; anions are shown in red) with a single aerolysin wt pore incorporated; the current through the pore is measured via Ag/AgCl electrodes. **b**, Typical single-pore *IV* curve where the current of the open pore is measured at different voltages consecutively. A slight rectification is visible and indicated through arrows at ±100 pA. Inset: the voltage trace that can be used to acquire such data. **c**, Absolute current over time traces at constant potential of a single aerolysin wt nanopore. The same pore is measured first at 100 mV (green) and then at −100 mV (red) for 240 s. Rectification is clearly visible as the absolute current is higher at −100 mV. At 100 mV, the measured current is constant, while at −100 mV, the current decreases spontaneously; this behaviour is referred to as gating. **d**, Left: 39 gating traces recorded for 1 single aerolysin wt pore at −160 mV, coloured by current, showing the diversity of gating states associated with different current levels. The overlay is a zoom-in of the area shaded in grey. After recording the pore in the gated state, a bias of 0 mV was applied to open the pore. Right: violin plot of gating (red) and open-pore (green) current levels measured in 4 different single pores (replicate experiments) at −160 mV. Each randomly offset point in the violin plot represents the average of the gating or open-pore current of one gating trace. The white error bar denotes ±1 standard deviation of the mean of the average closed-state currents. The large variation in closed-state current compared to open-pore current variation suggests a stochastic phenomenon. All experiments are done in 1 M KCl buffered to pH 6.2 with 10 mM phosphate.

To demonstrate the nonlinear responses of biological nanopores, we begin by examining the response of single aerolysin wild-type (wt) pores to a constant applied potential. Figure 1a illustrates our experimental set-up, where a nanopore is embedded into a lipid membrane separating two electrolytic solutions containing two silver/silver-chloride electrodes (‘Experiments’ in Methods).

When probed at different voltages for a few seconds at a time to obtain the current versus voltage (*IV*) curve, a small rectification is observed (Fig. 1b). We then applied a constant potential of both opposite signs for a few minutes to monitor pore stability (Fig. 1c). We observed that after a few tens of seconds, the negative current trace suddenly reduces by more than 90%. This abrupt decrease is the signature of gating^11^ and hinders analyte sensing applications where a stable baseline current trace is required^9,26^. Over the past decades, a number of experimental studies aimed at characterizing and understanding the gating phenomenon in β-barrel biological nanopores have been conducted and several hypotheses have been proposed, most convincingly conformational changes, among others^11,15–23^. However, no compelling evidence has been presented yet. Aerolysin, as well as other biological pores, thus exhibits two distinct nonlinearities. The first takes the form of an open-pore rectification, while the second is a delayed, sharp and polarity-dependent decrease in current, referred to as gating.

To investigate this behaviour further, we repeatedly recorded the same single aerolysin wt nanopore and found that, despite the current reducing to a discrete state or switching between several discrete states, these states are diverse in their average current. As shown in Fig. 1d, it is rare that the same state is observed in different gating events. Overall, we recorded four different pores—no clearly defined separated states emerged from this large sample (Fig. 1d). The low throughput of single-pore direct current (d.c.) measurements combined with the stochastic and diverse nature of gating makes this approach unsuitable for extensive characterization of the phenomenon. We thus rely on alternating current (a.c.) measurements to understand this behaviour.

Biological pores exhibit history-dependent conductivity as they switch from the open to the closed state after approximately 30 s in the displayed trace (Fig. 1c). In other words, they behave as resistors with an internal variable sensitive to the applied voltage. Such devices are called memristors (resistors with memory). Intensively studied in electronics^27–29^, memristive behaviour has recently been reported in solid-state^30–33^ and biological nanofluidic devices^20,21^. As is standard in memristor studies, we quantified the similarly nonlinear dynamics of our biological nanopores through a.c. measurements of the *IV* curves.

## Open-pore rectification and gating characterized with time-varying voltage

**Fig. 2:**
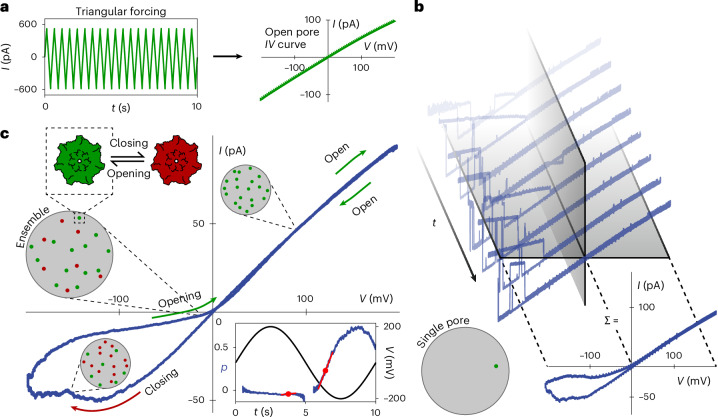
Methodology for the characterization of nonlinearities using time-varying voltage drop across single- and multi-pore membranes. **a**, Triangular forcing at 2 Hz, 200 mV, with 6 aerolysin wt nanopores used to extract the single open-pore *IV* curve. **b**, *IV* characteristics of a single aerolysin wt nanopore in the lipid membrane measured with sinusoidal potential (0.1 Hz). Fifty consecutive cycles are summed to retrieve the smooth hysteresis loop. **c**, Normalized *IV* characteristics of 26 pores in the same membrane showing the smooth hysteresis loop (0.1 Hz). Pores in the membrane close and open in a voltage-dependent manner, giving rise to the hysteresis loop. Arrows indicate the direction of the sweep. Inset: gating quantification. The solid blue line represents the closed-state probability, *p*, and the solid black line represents the sinusoidal voltage. The calculation of *p* is ill-defined around the origin, resulting in a discontinuous line in the probability plot, which did not affect our conclusions (‘Data analysis’ in Methods). The maximum slope indicated by the red lines and dots corresponds to *k*_X_ for both polarities, where *k*_X_ is the maximum closing rate. All experiments were conducted in 1 M KCl buffered with 10 mM phosphate to pH 6.2.

We used a periodic potential bias to measure and quantify both open-pore rectification and gating in a robust and rapid manner. For the open-pore rectification, triangular forcing was applied across a single pore at a frequency high enough for gating-free cycles to be observed (~Hz) (Fig. 2a). The rectification of a given pore is then quantified as a unitless value between −1 and 1 by the rectification factor *β* = (*I*_*+*_ − ｜*I*_*−*_｜)/(*I*_*+*_ + ｜*I*_*−*_｜), where *I*_*±*_ = *I*(*V* = ±100 mV). To investigate gating, we chose a low-frequency sinusoidal span to maximize the time spent at higher voltages and therefore the closed-state probability.

When this low-frequency voltage was applied, the single aerolysin wt pore switched between open and closed states in the previously described stochastic, voltage-dependent manner, the pore remained in the open state at positive biases and closed at negative applied biases. The stochasticity resulted in substantial variations of the *IV* curve at negative voltage between cycles (Fig. 2b). When 50 consecutive cycles of the same pore are averaged, the characteristic memristive hysteresis becomes visible, in the form of a loop in the *IV* curve (Fig. 2b). Analysing this stochastic phenomenon thus requires a large number of repetitions to extract its average behaviour. We found that measuring a single sweep of multiple pores in the membrane simultaneously (ensemble average) (Fig. 2c) results in the same response as the time average performed in Fig. 2b.

Contrary to previous interpretations^16^, this proves that gating is ergodic. Such ergodicity is further confirmed through phenomenological modelling and single pulse operation (Supplementary Section 3). Following this analysis, we normalize the ensemble conductance by the open-pore conductance to obtain *p*(*t*)—the time-dependent probability for the pores to be closed. The maximum derivative of *p*(*t*), here called *k*_X_, was used as an estimate for the rate of closing (Fig. 2c, inset, and ‘Data analysis’ in Methods), which we chose to quantify gating in various experimental conditions.

Overall, ensemble measurements offer three major advantages over single-pore measurements. They offer a higher throughput and a larger signal-to-noise ratio, and have a higher success rate since it is much easier to incorporate many pores in a membrane than just one. We now harness this technique to understand the role of pore lumen charge on ion transport in biological pores. To achieve this, through mutations, we tuned the lumen charge at specific locations inside the different pores, thus creating a mutant library. We focus mainly on aerolysin because of its constant lumen radius, even though our conclusion may be extended to other pores with variable radii.

## Open-pore rectification of aerolysin is directly driven by lumen charge

Mutations allow us to easily add, delete or change lumen charges at a resolution equal to the multimericity of the pore. We provide an extensive description of aerolysin’s lumen charge landscape in Supplementary Section 4.

**Fig. 3:**
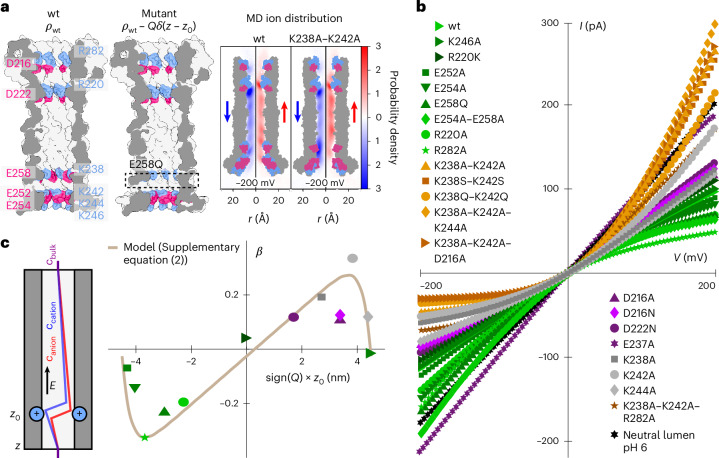
Characterization of open-pore current rectification using a wide library of aerolysin mutants. **a**, Left: sketches of aerolysin wt and mutant E258Q showing the deletion of a charged residue; positive charges are drawn in blue and negative charges are drawn in pink. Right: ion radial probability distributions (blue for K^+^, red for Cl^−^) measured on the MD simulations of aerolysin wt and mutant K238A–K242A at −200 mV. **b**, Open-pore *IV* curves of several mutants of aerolysin showcasing the different degrees of rectification tuned by the lumen charge (1 M KCl, 10 mM phosphate pH 6.2, 1 Hz). **c**, Left: schematic representation of mutant E258Q in the rectification model. The deletion of E258 that is negatively charged is represented as the addition of a fixed positive charge in the pore’s energy landscape, leading to ion accumulation downstream of the fixed charge. *E*, electric field. Right: rectification factor *β* as a function of the sign and position of the charge deleted after mutation. Solid curve: theoretical model (Supplementary equation (2)).

We mutated different combinations of these charged amino acids to change the charge distribution of the pore lumen and thus measured a library of 26 different aerolysin mutants (Supplementary Fig. 3). In what follows, we first focus on the consequences of mutations over the open-pore ionic transport by using high-frequency sweeps at low pore numbers. We then study the effect of mutations on the gating behaviour. We report in Fig. 3b*IV* curves measured with high-frequency sweeps for all our aerolysin mutants (separated curves can be found in Supplementary Fig. 3). A first observation is that modifying lumen charges has dramatic consequences for the shape of the open-pore *IV* curve. Convex and concave rectification can be observed, along with linear behaviour. Further, we find that the conductance of all mutants is below the bulk theoretical conductance of 2 nS by a factor of 2–8 (Supplementary Table 1). We attribute the reduced and nonlinear ionic conduction to the presence of energy barriers arising from the combination of lumen charge and nanometric confinement. Altogether, these results demonstrate that ion transport through aerolysin, such as α-HL^12,34^, is controlled by lumen charges and highly sensitive to its modifications. The effect of ionic rectification, also known as a nanofluidic diode, has been studied in many experimental and theoretical investigations in biological channels^35^ as well as solid-state nanofluidics^36–38^. However, while recent advances have enabled the fabrication of sub-nanometre pores in two-dimensional (2D) materials and angstrom slits^39^, solid-state nanofluidics has predominantly focused on relatively large channels (~10–100 nm in diameter) with spatially extended surface charges and geometrical asymmetries while smaller biological nanopores are explored mainly through simulations. Here we developed a theoretical framework to tackle the effect of individual, localized charge in a biological pore with a sub-nanometric radius. To rationalize this, we derived an analytical theory (detailed in Supplementary Section 1) that accounts for rectification and links its magnitude to the distribution of charge in the pore. In brief, we modelled aerolysin as a cylindrical pore with a distribution of surface charges *ρ* corresponding to charged residues (Fig. 3a). These charges define the potential energy landscape experienced by diffusing ions. When an electric field is applied, ions accumulate downstream of potential wells and upstream of potential barriers, and are depleted in opposite configurations (Fig. 3a and Supplementary Fig. 7). Depending on the distribution of lumen charges, which act as potential wells for counterions, this effect leads to an overall increase or decrease in the number of ions inside the pore, and likewise in conductance. We observe an excellent agreement between the model and experimental data (Fig. 3c). In particular, our model correctly predicts that the charged groups located near but not at the mouth of the pore play a key role in ionic conduction. This is because a charge that is placed at the mouth of the pore will modify counterion distributions mostly in the reservoir, rather than inside of the pore.

When removing all above-mentioned charged amino acids present in aerolysin wt, the resulting *IV* curve is almost perfectly ohmic (Supplementary Fig. 4). We can thus deterministically control the degree of rectification as well as completely suppress it, relying on rational, site-specific mutations. We also experimentally confirmed that variations in ionic transport are mainly controlled by charge modification rather than a change in pore diameter or hydrophilicity by observing that mutating lysines (K) (168.6 Å^3^) to alanine (A) (88.6 Å^3^), serine (S) (89.0 Å^3^) or the much larger glutamine (Q) (143.8 Å^3^)^40^ leads to nearly equivalent rectification (Supplementary Fig. 3). Having fully rationalized the relationship between lumen charge and open-pore ionic conduction, we now study the influence of lumen charges and their modification on gating.

## Gating behaviour is controlled by pore charge

**Fig. 4:**
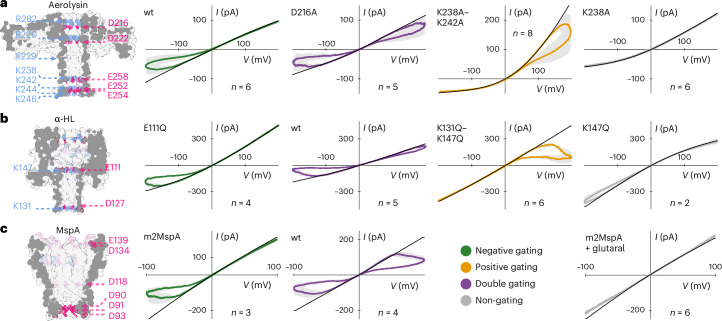
Lumen charge-dependent gating behaviour measured with different aerolysin, α-HL and MspA mutants. **a**–**c**, Gating behaviour measured with different aerolysin (**a**), α-HL (**b**) and MspA (**c**) mutants, showcasing the great tunability of gating by changing lumen charge. Mutants gating at negative potentials shown in green, at positive potentials in orange, mutants showing low levels of gating in grey and mutants that gate at both polarities in purple. Each plot shows the open-pore *IV* curve in black, showing the differences in rectification. Further, each plot shows ±1 standard deviation of the mean over the number of experiments *n* plotted as a grey area. All experiments were conducted in 1 M KCl buffered to pH 6.2 with 10 mM phosphate. Sinusoidal forcing was applied at 0.1 Hz and 200 mV amplitude (100 mV for MspA wt and m2MspA).

**Extended Data Fig. 1:**
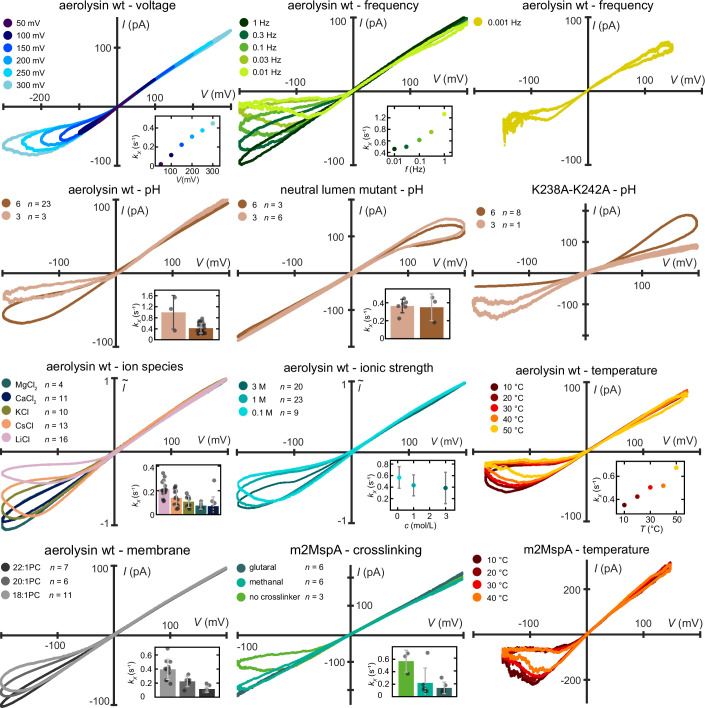
Gating as a function of voltage, frequency, pH, ion species, ionic strength and temperature. Voltage, frequency and temperature dependencies are measured in the same experimental conditions and display of the number of experiments, *n*. The ionic species, ionic strength are recorded with 150 µm, aperture MECA-chips and the ionic species measurements are buffered with 10 mM MES instead of phosphate to a pH of 6.35 ± 0.15 and are recorded at 0.03 Hz. Unless otherwise specified all recordings are done at 0.1 Hz, 1 M KCl, pH 6.2, 25 °C. In the inset the computed closing rate is shown, with error bars that show ± 1 standard deviation of the mean over *n*. The neutral lumen mutant exhibits the following mutations: K238A-E258N-K242S-E254N-K244S-E252N-R282A-D216N-R220A-D222N-K246A-D209N-R288A.

We now examine the gating behaviour of our mutant library. We show in Fig. 4a–c the *IV* curve obtained for several mutants of aerolysin, α-HL and MspA using low-frequency sinusoidal sweeps. We can observe that, depending on the mutations, gating occurs at positive, negative or both polarities, or is largely absent. Gating appears to be additive: for example, aerolysin wt shows negative gating; the two mutations, K238A and K242A, individually lead to a suppression of gating, and collectively (K238A–K242A) change the gating to positive (Fig. 4a and Supplementary Fig. 8). This finding suggests that the underlying gating mechanism remains the same for all mutants, and that the lumen charges individually contribute to the stability of the closed state under a given voltage, tuning the kinetics (for example, closing rate) of gating. Taken together with previous observations^3,25,41–43^, this demonstrates both the universality of this phenomenon in β-barrel-forming nanopores and its sensitivity to local charge variations. To further back the phenomenon’s universality, we confirmed the previously observed increase in gating with decreasing charge carrier concentration^19,44^, pH^18,44–46^ and temperature^47,48^ (Extended Data Fig. 1). As for rectification, we verified that gating is strongly dependent on the charge but not on the size or hydrophilicity of the exchanged amino acids (Supplementary Fig. 9 and Supplementary Table 1). Since both phenomena share a common dependency, we explore their correlation.

**Fig. 5:**
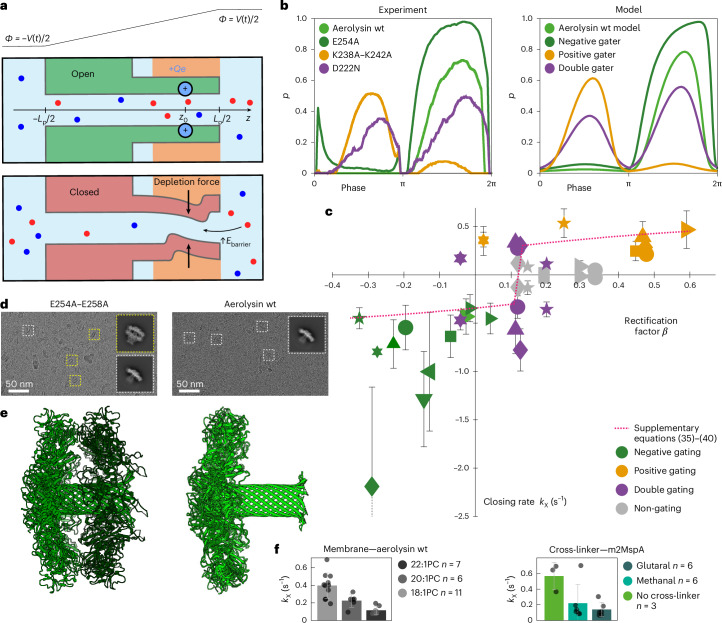
Evidence in support of a structural deformation responsible for gating. **a**, Sketch illustrating both states of the model. The pore exhibits mechanical bistability induced by depletion forces: when the pore diameter fluctuates below a certain threshold, ions are expelled from the pore, causing it to collapse partially. This effect is promoted by the dissociation of counterions from lumen charges under an applied voltage. **b**, Closed-state probability plotted against the phase of the voltage for aerolysin wt and four representative mutants obtained from experiments (left) and the biophysical model (right) (see Supplementary Section 2 for details). The model accurately predicts whether the given mutant is a positive, a negative or a double gater. It also predicts that the E254A mutant gates stronger than aerolysin wt. **c**, Scatter plot of the pH response and all 26 aerolysin mutants’ closing rates versus rectification factor *β*. Pores that show low gating or gating at both polarities are represented twice. For pores that show gating only in the negative or positive quadrant, only one value is shown. The error bars show ±1 standard deviation of the mean over the number of experimental replicas *n*, and each symbol represents a different mutant (Supplementary Fig. 11). Dotted line: theoretical prediction based on models of rectification and gating (Supplementary Section 2). **d**, Cryo-EM images of E254A–E258A mutant (left) and aerolysin wt (right). Boxes delimited with dashed lines show corresponding 2D classes of the two pore populations; the box size is 290 angstrom. **e**, Stack of a mutant pore (green) and a mutant prepore (dark green) (left) and alignment of the structures of wt (light green) and the E254A–E258A (9GXJ) mutant (green) of a fully formed pore (right). **f**, Closing rates of aerolysin wt for different membrane thicknesses (left) and of m2MspA and m2MspA cross-linked with glutaral and methanal (right) (1 M KCl, 10 mM phosphate, pH 6.2, 0.1 Hz). The error bars show ±1 standard deviation of the mean over the number of experimental replicas *n*.

For aerolysin, we observed a correlation between closing rate, obtained from low-frequency sweeps, and rectification factor *β*, obtained from high-frequency sweeps (Fig. 5c). This means that gating is most likely to occur at the polarity where the open-pore current is highest (Figs. 1c and 5c). The fact that our rectification model singles out lumen charges as the reason for rectification together with the observed correlation of the closing rate and *β* across a wide range of mutants further points to the key role of lumen charge in gating.

While tuning of the lumen charge affects gating behaviour very predictably in aerolysin, α-HL and MspA (Fig. 4a–c), the aerolysin mutant with a neutral lumen exhibits polarity-dependent gating (Supplementary Fig. 9). This strongly suggests that additional charges, such as those placed outside the lumen (for example, K229), also play a role in gating. A non-charge-related mechanism is not likely precisely because of the polarity dependence of the gating of the neutral lumen mutant exhibiting linear conductance. Overall, this suggests that the pore exhibits some mechanical bistability even without lumen charges, but the latter strongly control this effect.

## Gating can be explained by a voltage-induced conformational change

A reversible change of lumen conformation triggered by voltage and controlled by lumen charge as well as mechanical properties has been previously hypothesized for the studied pore-forming proteins^20–22^ and was demonstrated for structurally different voltage-gated ion channels^10,49^. In some cases, gating was attributed to the motion of flexible and charged elements within the pore^43^; however, gating could still be observed when these groups are deleted^50^. This suggests that gating could emerge from the structure and dynamics of the β-barrel itself.

**Extended Data Fig. 2:**
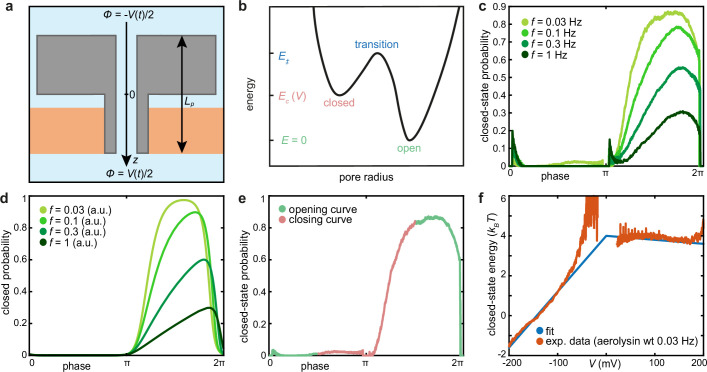
Constraining the biophysical model. **a**. Geometry of the system. **b**. Two-state toy model of gating: the system is transitioning between states corresponding to different values of the pore radius, with transition rates given by the states’ energies. c. Evolution of closed state probability with frequency. Experiments performed on aerolysin wt, amplitude 200 mV, 25 °C, 1 M KCl, pH 6.2 buffered with 10 mM phosphate. **d**. Effect of frequency in the two-state toy model, suggesting that only the energy of the closed state varies with the external voltage. **e**, **f**. Determination of the energy of the closed state, from the opening branch of the probability-voltage curve.

**Extended Data Fig. 3:**
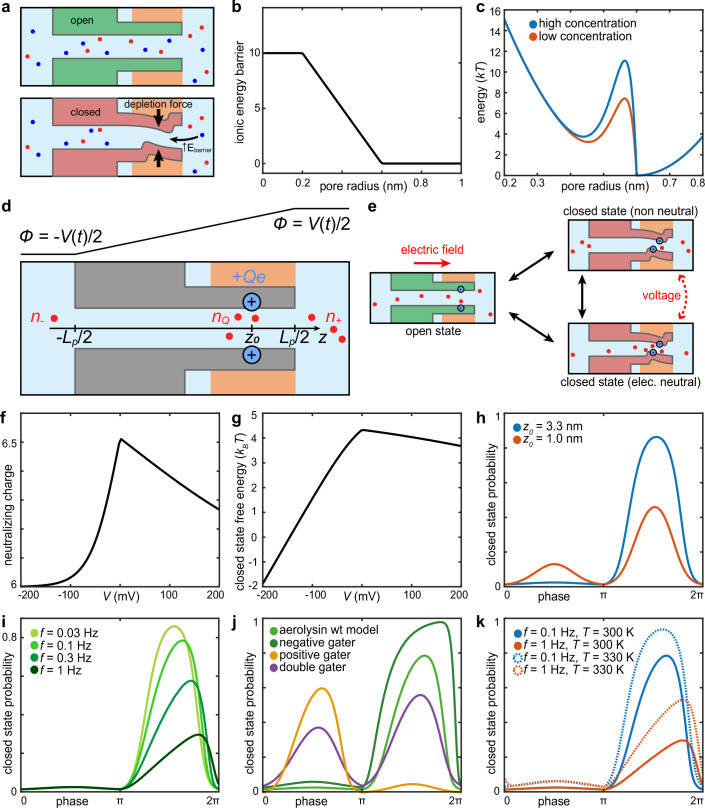
Model of ionic gating in aerolysin. **a**. Schematic representation of depletion interactions. In the open state, the pore is relatively wide and permeable to ions. In the closed state, ions face an energy barrier owing to the smaller radius, resulting in depletion interactions. **b**. Simplified energy profile for the ionic energy barrier *μ*_ex_. **c**. Energy landscape of the pore under zero voltage, owing to depletion interactions (orange curve, concentration 1 M; blue curve, concentration 1.2 M). **d**. Parametrization of the gating model **e**. Schematic representation of the model’s derivation. **f**-**k**. Results of the model, showing the number of counterions neutralizing the fixed lumen charge (**f**), the free energy of the closed state (**g**) and the closed state probability for various lumen charge positions (**h**, **j**), voltage frequency (**i**) and temperature (**k**). All parameters are detailed in the text.

On the basis of experimental findings thus far, we build an analytical model for gating in aerolysin (Extended Data Figs. 2 and 3), detailed in Supplementary Section 2. In brief, the flexibility of the β-barrel allows radius fluctuations that modulate ion’s entry barriers. This results in a structural bistability, where ionic depletion and pore collapse reinforce each other. External fields enhance this effect by partially breaking electroneutrality under strong confinement, rendering lumen charges sensitive to voltage polarity. We quantified this gating process as a function of applied voltage and charge distribution (Fig. 5a,b). This model recapitulates all the key experimental features of gating such as gating polarity inversion in certain mutants, or the existence of weak positive gating even in strong negative gaters, as well as the effects of ionic strength (Extended Data Figs. 1 and 3c) and temperature (Extended Data Figs. 1 and 3k).

Lastly, combining our models of rectification and gating, we can rationalize the correlation observed between these two effects. Both effects are stronger when the electric field is directed from lumen charges towards the closest entrance of the pore, therefore dragging counterions over a substantial portion of the channel. Plotting the theoretical closing rate versus the rectification factor *β*, we find good overall agreement with experimental data (Fig. 5c, red dashed line). The graph features a vertical line, where gating and rectification seem to decouple: this corresponds for the most part to mutants with no net lumen charge (exhibiting only pairs of opposite lumen charges, which contribute to rectification but not to gating), and mutants with approximately symmetrical lumen charges of the same sign (causing gating in both polarities, but contributing only weakly to rectification). Overall, these results highlight how charge location can affect nonlinear ion transport in biological nanopores across both fast and slow timescales.

We also perform several control experiments corroborating the hypothesis of voltage-induced structural deformations. When comparing cryo-EM structures of aerolysin wt and the strongly gating E254A–E258A mutant, we find that around 44% of the E254A–E258A particles appear in dimeric pore stacks formed by one mature aerolysin pore within an aerolysin prepore (Fig. 5e), similar to what was previously found with a disulfide-cross-linked K246C–E258C mutant^51^. This indicates that while aerolysin wt univocally forms a mature pore β-barrel in multiple membrane-mimicking environments^52^, the E254A–E258A mutant shows a much lower efficiency for β-barrel formation as roughly half of the particles observed are trapped in the prepore conformation where the β-barrel is not yet formed. This suggests that these mutations impact the mechanical stability of the pore. Another hint is the dependency of gating with membrane thickness (Fig. 5f and Extended Data Fig. 1). Membrane thickness and composition are known to influence the dynamics of membrane proteins^53^, and have been implicated in gating^54,55^. By changing the lipid tail length, we find that gating decreases with increasing membrane thickness. The change in thickness leads to a change in the lipid–protein contact area, which leads to a shift in the forces acting on the protein through a difference in hydrophobic mismatch, pressure and tension, as well as potential steric hindrance with the above-membrane parts of the nanopore. The observation that gating is membrane dependent points to the transmembrane region shared by all β-barrel nanopores, as the active site of the mechanism. To further verify the hypothesis of a conformational change, we cross-linked the highly gating m2MspA pore with glutaral or methanal to increase its mechanical stability (Fig. 5f and Extended Data Fig. 1). We find that such cross-linked pores do not gate while maintaining the same rectification and conductance (Supplementary Table 2) as the untreated m2MspA.

Mutating charged residues in the pore lumen not only alters the electrostatic environment but also disrupts the hydrogen-bond network, salt bridges and other intermolecular forces, leading to changes in the mechanical stability of the pore^18^. This intricate interplay between lumen charge and mechanical deformation within nanometric confinement helps explain why characterizing and molecularly understanding gating remains so challenging. Nevertheless, the overwhelming uniformity with which these β-barrel nanopores respond to voltage, frequency, temperature, ionic strength, pH and lumen charge distribution strongly indicates a common underlying mechanism. Importantly, while our work focused on multimeric β-barrel nanopores, all comparable results reported for monomeric β-barrel channels are consistent with our observations, supporting the conclusion that this phenomenon extends across both classes. More broadly, we expect it to be generally applicable to any pore with a radius around 1 nm, although other pore-specific mechanisms may also contribute to gating.

## Synaptic plasticity emulated with engineered biological nanopores

**Fig. 6:**
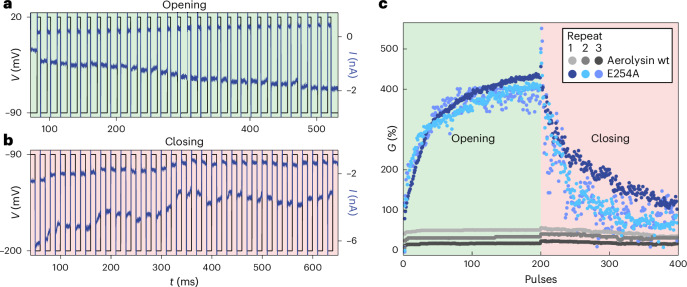
Synaptic dynamics with biological nanopores. **a**, Synaptic potentiation, through repeated voltage pulses, is used to open the ensemble of nanopores in the membrane. Pulses (110 mV) with a duration of 5 ms are used. **b**, Synaptic depression through repeated voltage pulses, used to close the ensemble of nanopores in the membrane. Pulses (−110 mV) with a duration of 10 ms are used. For both **a** and **b**, the waiting time between pulses was 10 ms and the first 30 pulses are shown. The baseline was fixed at −90 mV to minimize closing or opening of the pores. **c**, Conductance evolution during potentiation and depression. The conductance normalized by the closed-state current is shown for three repeat experiments of aerolysin wt and E254A. The overshooting when switching from opening to closing is due to the rectification.

To demonstrate the implications of our findings in the context of ionic bio-computing^30^, we implement a synaptic behaviour using E254A. This aerolysin mutant is chosen for its strong gating rate and on/off ratio (Fig. 5b and Supplementary Table 1). We also study weakly gating aerolysin wt as a benchmark showing little synapse-like plasticity. The membrane’s conductance can be increased/decreased sequentially (potentiation/depression) upon the application of positive/negative voltage pulses (Fig. 6a–c). The pores are thus ‘learning’ upon repeated stimulation such as a biological synapse. The procedure and performances are detailed in Supplementary Section 10. By relying on protein mutation, it is therefore possible to engineer nanofluidic synapses with tailored plasticity.

## Conclusion

In this work, we demonstrate that key aspects of biological ion transport can be rationalized using a nanopore characterization approach based on alternating current, offering new insights into voltage-dependent transport processes. We show that the heterogeneous lumen charge controls two distinct nonlinearities: open-pore rectification and gating. Our work encompasses a comprehensive database of mutants, elucidating this phenomenon beyond what has been demonstrated in numerous previous studies^10,20,22^. In light of these findings, we built detailed biophysical models of rectification and gating. Notably, we propose that gating results from ionic depletion interactions that lead to a partial deformation of the β-barrel. Then, we show that the collapsed state can be stabilized by the external voltage through a dissociation of lumen charge–counterion pairs. Overall, we provide a plausible and detailed explanation that accounts for the observed nanopore behaviour across a broad range of experimental conditions and control settings. Direct experimental evidence for the physical origin of this extensively studied phenomenon remains challenging to obtain. Nonetheless, the conformational dynamics of the β-barrel that we uncovered calls for confirmation using techniques such as single-molecule Förster resonance energy transfer (FRET), and our theoretical developments could be supplemented with enhanced sampling approaches to simulate the pore dynamics.

Our work provides guidelines for nanopore engineering in sensing applications. Furthermore, we show that rectification and gating can be used to generate iontronic components such as resistors, diodes or memristors with a tuning resolution reaching the physical limit of 1 single charge. While the construction of multi-component ionic computing systems is still at its infancy^32^, our synaptic plasticity results show that such networks could potentially use biological building blocks with dimensions similar to state-of-the-art electronic transistors.

## Methods

### Material

We purchased 1,2-diphytanoyl-sn-glycero-3-phosphocholine (DPhPC), 1,2-di-*O*-phytanyl-sn-glycero-3-phosphocholine (DoPhPC), 1,2-dimyristoleoyl-sn-glycero-3-phosphocholine (14:1_Δ9-Cis_PC), 1,2-dipalmitoleoyl-sn-glycero-3-phosphocholine (16:1_Δ9-Cis_PC), 1,2-dioleoyl-sn-glycero-3-phosphocholine (18:1_Δ9-Cis_PC), 1,2-dieicosenoyl-sn-glycero-3-phosphocholine (20:1_cis_PC), 1,2-dierucoyl-sn-glycero-3-phosphocholine (22:1_cis_PC) dissolved in chloroform from Avanti Polar Lipids, *n*-octyltetraoxyethylene (OTOE) from Bachem, plasmids from Genscript, Turbonuclease from Lucerna-Chem AG, HEPES from Chemie Brunschwig AG, 0.45 µm Rotilabo PVDF syringe filter from Carl Roth GmbH & Co. KG, isopropyl β-*d*-1-thiogalactopyranoside (IPTG) from Huberlab, defibrinated sheep blood from Rockland Immunochemicals, HiPrep 26/10 Desalting column, HisTrap HP column and PD-10 desalting columns packed with Sephadex G-25 resin from Cytiva, and all other chemicals from Merck. All electrolyte solutions used in bilayer experiments were buffered with 10 mM phosphate and filtered through 0.22 µm pore size PES vacuum filters from VWR International. We used purified water (18.2 MΩ) from a Milli-Q water purifying system. Ag/AgCl wire electrodes were made by immersing one side of high-purity silver wire pieces in bleach for 1 h and subsequent washing with water. Lipids were aliquoted at 0.2 mg per glass vial, chloroform was evaporated under vacuum, and vials were subsequently closed with polytetrafluoroethylene (PTFE) lined caps in an argon glove box and stored at −20 °C to avoid oxidation. Lipids were dissolved freshly in octane or pentane at 10 mg ml^−1^.

#### Aerolysin purification

The clone of the full-length aerolysin wt protein (in the pET22b vector with a C-terminal hexa-histidine-tag (His-tag)) was kindly provided by the van der Goot laboratory at EPFL. The mutants were generated by Genscript using the wt plasmid as a template. The aerolysin protein was produced as previously described^14^. In brief, plasmids were transformed into BL21 (DE3 Plys) *Escherichia coli* cells by heat shock. Cells were grown to an optical density of 0.6–0.7 at 600 nm in LB media. Protein expression was induced by the addition of 1 mM IPTG and subsequent growth overnight at 18 °C. Cell pellets were resuspended in aerolysin lysis buffer (500 mM NaCl, 50 mM tris, pH 7.4), mixed with cOmplete Protease Inhibitor Cocktail and then lysed by sonication on ice. Turbonuclease solution was added to the resulting suspension, which was then centrifuged (20,000 × *g* for 30 min at 4 °C). After syringe filtration over a 0.45 µm filter, the supernatant was applied to a HisTrap HP column previously equilibrated with aerolysin lysis buffer. The protein was eluted as a monomer with a gradient of 30 column volumes of aerolysin elution buffer (500 mM NaCl, 50 mM tris, 500 mM imidazole, pH 7.4). Aerolysin-containing fractions were then buffer-exchanged to aerolysin final buffer (20 mM tris, 150 mM NaCl, pH 7.4) using a HiPrep Desalting column. The purification of the selected fractions was confirmed by sodium dodecyl sulfate–polyacrylamide gel electrophoresis. Aerolysin was concentrated to 0.5 mg ml^−1^ and stored at −20 °C. The activation of aerolysin to undergo conformational changes and enable heptamerization is achieved by incubating it with trypsin in agarose at a ratio of 1:4 (trypsin/pore, v/v).

#### α-HL purification

Coding sequences for all α-HL mutants were custom synthesized, optimized for *E. coli* expression and inserted into the pET28-b vector between the NcoI and XhoI cleavage sites, such that the resulting proteins carry a C-terminal His-tag for later purification. Plasmids were transformed in BL21 (DE3) cells by heat shock transformation. The cells were grown in LB medium at 37 °C until an optical density of 0.6–0.7 at 600 nm at which point protein expression was induced by the addition of 0.1 mM IPTG, followed by subsequent overnight growth at 18 °C. Cell pellets were resuspended in α-HL lysis buffer (500 mM NaCl, 20 mM HEPES, 1% Triton X-100 (v/v), pH 8.0), mixed with cOmplete Protease Inhibitor Cocktail and then lysed by using sonication on ice. Turbonuclease solution was added to the resulting suspension, which was then centrifuged (20,000 × *g* for 60 min at 4 °C). After syringe filtration over a 0.45 µm filter, the supernatant was applied to a HisTrap HP column previously equilibrated with α-HL lysis buffer. The column was then washed with 3 column volumes of α-HL lysis buffer followed by a wash with 10 column volumes of α-HL wash buffer (500 mM NaCl, 20 mM HEPES, 5 mM imidazole, pH 8.0). The α-HL heptamers were then eluted with a linear gradient of α-HL elution buffer (500 mM NaCl, 20 mM HEPES, 500 mM imidazole, pH 8.0). α-HL pores were not buffer-exchanged to a final buffer but rather stored in the ratio of α-HL wash buffer and α-HL elution buffer that they eluted in. Peak fractions were analysed by SDS–polyacrylamide gel electrophoresis and only the fractions with the α-HL heptamer were retained for subsequent planar lipid bilayer experiments.

#### MspA purification

All MspA mutants were synthesized and cloned into pET28b vectors (NcoI/XhoI, C-terminal His-tag), transformed into BL21 (DE3). Protein expression was induced (0.1 mM IPTG, OD600 0.6, overnight, 18 °C). Pellets were resuspended in MspA lysis buffer (100 mM sodium phosphate, 0.1 mM EDTA, 150 mM NaCl, 0.5% Genapol X-80, pH 6.5), with protease inhibitors, lysed by sonication on ice, boiled (60 °C, 10 min), treated with Turbonuclease, chilled on ice (10 min) and then centrifuged (20,000 × *g*, 60 min, 4 °C). After filtration, the supernatant was purified using a HisTrap HP column, washed with lysis and wash buffers and eluted with a gradient of MspA elution buffer. Fractions were buffer-exchanged to MspA final buffer (500 mM NaCl, 20 mM HEPES, pH 8.0, 0.6% OTOE) using PD-10 columns.

Cross-linked pores were prepared as follows. MspA wt or m2MspA (100 μl) was mixed with 10 μl of 8% (v/v) glutaral in water to a final concentration of 0.8% glutaral and incubated for 10 min before use. For pores cross-linked with methanal, 100 μl of pore solution were mixed with 5 μl of 20% (v/v) methanal in water to a final concentration of 1% (v/v) methanal and incubated for 10 min before use.

### Experiments

#### Planar lipid bilayer experiments

Unless stated otherwise, an Orbit Mini (Nanion Technologies) with 50 µm aperture MECA-4 chips (Ionera Technologies) was used for all experiments. Bilayers were painted by dipping, pipette tips in 10 mg ml^−1^ DPhPC in octane and subsequent dabbing on Kimwipes, so that all visible liquid was removed from the pipette tip. The pipette tip was then immersed in the electrolyte solution-filled MECA-4 chip and placed over one of the four apertures, and a bubble was pushed in and out of the pipette tip until a membrane was formed. This usually required several tries, exchanging lipid-primed pipette tips frequently. For all open-pore *IV* curve recordings, we pretreated PTFE films (Eastern Scientific LLC) with a single aperture with a diameter of 50 μm by pipetting 1 μl of 1% (v/v) hexadecane in hexane onto both sides of the aperture of the PTFE film. We mounted the PTFE film in a PTFE chamber using high-vacuum grease (Dow Corning). The PTFE film separated two compartments containing aqueous electrolyte, which were only connected by the aperture in the PTFE film over which the bilayer was formed. We formed planar lipid bilayers across the aperture using a technique previously described^56,57^. In brief, we added electrolyte solution to both compartments so that the level was below the aperture and pipetted 3 μl of DPhPC dissolved in pentane onto the surface of the electrolyte solution. After the pentane evaporated, a lipid monolayer formed at the air–aqueous interface. We raised and lowered the electrolyte solution until we measured a very small baseline current (−3 pA < *I* < 3 pA), indicating that a membrane had formed. All single-pore d.c. experiments and the experiments with sine wave frequencies at and below 0.001 Hz were measured in painted bilayers formed on self-made PTFE/FEP supports made from Dual-Shrink tube (Zeus) using a tungsten needle, sharpened from a welding electrode. Supports were pretreated by pipetting 1 µl DoPhPC in hexane (2 mg ml^−1^) onto the support, blowing through the support from the backside with an air-filled syringe to clear it from the lipid solution. This pretreatment was repeated two times with short drying cycles in a desiccator under vacuum. The bilayer was painted from pure DoPhPC wetted with hexadecane with a two-haired fine paintbrush. We verified the stability (absence of leak currents; expected noise and capacitance level) of all bilayers by briefly applying transmembrane voltages of up to 200 mV. The current was measured with an Axopatch 500B amplifier (Molecular Devices) through Ag/AgCl pellet electrodes (World Precision Instruments) or Ag/AgCl wire electrodes. Ag/AgCl wire electrodes were chlorinated by immersing coiled silver wire in bleach for 1 h before rinsing thoroughly with demineralized water. After use, electrodes were stripped from silver chloride with ammonium hydroxide and re-chlorinated. We assembled experimental set-ups inside Faraday cages. Voltages were applied externally through NI PXI-8336 and PXI-4461 controlled by in-house LabVIEW software; both applied voltage and resulting current were measured. We carried out all experiments at room temperature (23–25 °C) and tested all set-ups with different model cells to confirm proper functionality of the amplifiers. We cleaned the set-ups by rinsing all parts that came in contact with the electrolyte, nanopores and lipids with several cycles of demineralized water and isopropyl alcohol. We subjected PTFE cells and films to additional cleaning with chloroform to remove vacuum grease.

#### Cryo-EM

The aerolysin E254A–E258A sample in SMALP and cryo-EM grids were prepared as described previously^52^. The dataset was collected at the Dubochet Center for Imaging using the 300 kV TFS Titan Krios G4 equipped with a Cold-FEG and Falcon 4i detection. The dataset was collected in electron counting mode. The Falcon 4i gain references were measured before starting the data collection. The data collection was performed using the TFS EPU software packages. Movies were recorded at a nominal magnification of 120,000×, corresponding to 0.658 Å per pixel with defocus values ranging from 0.8 to 1.7. The exposure dose was set to 50 e Å^−^^2^. The datasets were processed in CryoSPARC^58^ (Supplementary Fig. 14) The reported resolutions are based on the gold-standard Fourier shell correlation (FSC) = 0.143 criteria^59^ and local-resolution variations were estimated using CryoSPARC. For the model building, the aerolysin wt (9FM6) and the post-prepore (5JZW) structures were used as initial models, which were fit into the cryo-EM map using Phenix^60^, manually adjusted using Coot^61^ and refined in Rosetta^60^ and Phenix. In the E254A–E258A pore structure (9GXJ) residues 14–23 and 246–251 and in the post-prepore (not quasi-pore) structure (9IGN), residues 190–304 were not built owing to a lack of density in these areas. The details on the processing and refining process and the EMDB IDs can be found in Supplementary Table 3.

### Simulations

The systems for atomistic MD simulations were prepared with the CHARMM-GUI^61^ website, using the structure of heptameric aerolysin wt available in the PDB with ID 5JZT, onto which the desired mutations were crafted using PyMOL. The protein heptamers were placed in the DPhPC membranes (CHARMM’s lipid name PHPC) using PPM 2.0 through the CHARMM-GUI interface. The systems were further prepared (solvated and neutralized) with standard CHARMM-GUI procedures and parameters in 1 M KCl. The systems were parameterized using CHARMM36m^62^ for the protein components and the corresponding version of TIP3P water, plus the standard CHARMM parameters available for DPhPC lipids. After standard minimization and equilibration procedures with standard parameters and restraint strengths using Gromacs 2022, we ran the production MD simulations applying the indicated voltages using the same MD engines applying semi-isotropic pressure coupling to 1 atm, a temperature of 298 K, 2 fs integration steps, LINCS-based restraints on hydrogens and PME electrostatics with 12 Å cut-off. Restraints on the Cα atoms were also applied allowing sampling the effect of side-chain-contributed electrostatics on ion currents without interference from structural perturbations that might be unrealistic given the limited resolution of the starting PDB structure and the high applied voltages. Simulations were extended for around 400 ns; and for all subsequent analyses, only the last 280 ns of the production phase was used. These analyses were carried out using standard VMD commands and custom scripts as detailed below.

The ionic flux at a given voltage bias was extracted from whole-atom simulations using a previously published method^63^. In brief, the instantaneous ionic current at time *t* was

1 $$I\left(t\right)=\frac{1}{\Delta t{L}_{{\rm{p}}}}\mathop{\sum }\limits_{\mathrm{ion}=1}^{{N}_{\mathrm{ion}}}{Q}_{\mathrm{ion}}[{z}_{\mathrm{ion}}(t+\Delta t)-{z}_{\mathrm{ion}}(t)]$$

where Δ*t* = 0.2 ns is the time gap between consecutive pairs of simulation snapshots written during production, *L*_p_ is the length of the pore β-barrel (9 nm for aerolysin), *N*_ion_ is the number of ions inside the pore lumen, and *Q*_ion_ and *z*_ion_ are the charge and *z*-coordinate of ion, respectively. The average current at a given voltage bias was computed by applying a linear regression to the cumulative sum of *I*(*t*).

### Data analysis

#### Alternating voltage traces

In the general case, assuming only two conductance states, the expected value for the conductance of an ensemble of gating pores *G*(*t*) is given by equation (2):

2 $$G(V,t)={N}_{{\rm{p}}}[(1-p(t)){G}_{{\rm{O}}}(V)+p(t){G}_{\mathrm{X}}(V)]$$

where *N*_p_ is the number of pores, *p*(*t*) is the time-dependent probability for the pores to be closed and *G*_O/X_(*V*) refers to the single-pore voltage-dependent conductance in the open/closed state, respectively.

Given the 2-state assumption *G*_X_(*V*) = *εG*_O_(*V*), the expected ensemble current, *I*(*t*), is

3 $$\frac{I(t)}{{I}_{{\rm{O}}}(t)}=\frac{G(t)}{{N}_{{\rm{p}}}{G}_{0}(t)}=1-p(t)(1-\varepsilon )$$

where *I*_O_(*t*) = *N*_p_*G*_O_(*t*)*V*(*t*) is the open-pore current obtained from a.c. measurements at high frequency (where the pore remains open for a full cycle (Fig. 2b)). Relying on single-pore measurements (Fig. 1d) where the gated state conducts 14% of the open-pore current on average, we assume *ε* = 0.14. Thus, from a recording of the ensemble current response to an alternating voltage, the frequency-dependent closed-pore probability could be estimated as

4 $$p\left(t\right)=\frac{1}{1-\varepsilon }\left(1-\frac{{I}_{\mathrm{ens}}\left(t\right)}{{I}_{{\rm{O}}}\left(t\right)}\right)$$

where *I*_ens_(*t*) is the average measured ensemble current of multiple a.c. cycles in the same ensemble. Note that for each cycle, the ensemble current was normalized by the estimated number of pores in that cycle, *N*_p_. To find the number of pores, a voltage window around *V* = 0 where no gating took place was predefined, and the current in this window was fitted to the *IV* curve in the same window using a linear regression, the slope of which gave *N*_p_. In all figures, the plotted current traces $\frac{{I}_{\mathrm{ens}}\left(t\right)}{{I}_{{\rm{O}}}\left(t\right)}$ are an average of *n* experimental replicates and each replicate experiment is a weighted mean of the cycles in that experiment (weighted by the number of pores in each cycle).

To compare gating strengths of different ensembles, an estimate of an ensemble’s closing rate was made. Supplementary Fig. 1 shows that when the pores start closing above a critical voltage *V*_c_, the opening rate is zero. Therefore, when the closing rate is at its maximum, the opening rate is negligible. Further, assuming that pores are closing fast, most pores are still open ($p(t)\approx 0$) when the closing rate is maximum. When these two conditions are met, $\frac{{\rm{d}}p(t)}{{\rm{d}}t}$ corresponds to the closing rate in the two-state gating description that governs pore opening and closing (Supplementary equation (41)). Thus, throughout a cycle, the estimate for the closing rate, *k*_X_ in Hz, was chosen to be the maximum of $\frac{{\rm{d}}p(t)}{{\rm{d}}t}$. In all figures, the plotted estimates *k*_X_ are an average of *n* experimental replicates.

### Statistics and reproducibility

In a.c. experiments, different mutants would always gate reproducibly regarding their polarity dependence. The closing rates of different mutants are also reproducible despite having a spread that we think is largely due to slight variations in the formed membrane (Supplementary Section 8). We performed 3–23 successful replicates each for all a.c. experiments apart from aerolysin mutants E258A (running out of pore) and K238A–K242A at pH 3 (membrane stability at pH 3) and α-HL mutant K147Q. Voltage and frequency dependencies were always consistent, and temperature dependencies and synaptic dynamics were confirmed three times. A single replicate comprises an average of minimum 1 to in the large majority several tens of pores in a single membrane. Each replicate comes from a different membrane. Further, the used MECA chips are cleaned between replicates and around 100 MECA chips were used in the study. We excluded data after a membrane broke and was reformed in case the gating behaviour after reformation changed because when the membrane is reformed, homogeneity of pore directionality is not ensured any longer. We excluded data where pores were incorporating into the membrane or where the membrane itself was unstable since a largely stable baseline current is needed. We excluded data where the open-pore *IV* curve of the respective pore did not fit the non-gating part of the gating *IV* curve since this indicates pores inserted in both directions. In d.c. gating experiments, we consistently saw the same stochasticity and spread of gating states across 4 different repeats with around 40 observations in each replicate. Cryo-EM results of E254A–E258A were repeated twice to confirm the result. Although seven different people independently acquired the electrophysiology data, the investigators were not blinded to allocation during experiments and outcome assessment. No statistical method was used to predetermine sample size. The experiments were not randomized.

## Online content

Any methods, additional references, Nature Portfolio reporting summaries, source data, extended data, supplementary information, acknowledgements, peer review information; details of author contributions and competing interests; and statements of data and code availability are available at 10.1038/s41565-025-02052-6.

## Supplementary information

Supplementary Information Theory notes, Supplementary Figs. 1–15 and Tables 1–3.

## Data Availability

All data that support the findings of this study are available within the article and its Supplementary Information. Source data are available via Zenodo at 10.5281/zenodo.17200775 (ref. ^64^). Cryo-EM data for aerolysin can be accessed through the EMDB with the code EMD-51664 for E254A–E258A and EMD-52853 for post-prepore and quasipore.

## Code availability

All data processing codes, simulation and modelling codes are available at https://github.com/lukasvandenheuvel/Biomemristors.
